# Mutual Exclusivity of Hyaluronan and Hyaluronidase in Invasive Group A Streptococcus*[Fn FN2]

**DOI:** 10.1074/jbc.M114.602847

**Published:** 2014-09-29

**Authors:** Anna Henningham, Masaya Yamaguchi, Ramy K. Aziz, Kirsten Kuipers, Cosmo Z. Buffalo, Samira Dahesh, Biswa Choudhury, Jeremy Van Vleet, Yuka Yamaguchi, Lisa M. Seymour, Nouri L. Ben Zakour, Lingjun He, Helen V. Smith, Keith Grimwood, Scott A. Beatson, Partho Ghosh, Mark J. Walker, Victor Nizet, Jason N. Cole

**Affiliations:** From the a Department of Pediatrics,; e Systems Biology Research Group,; h Department of Chemistry and Biochemistry,; m Skaggs School of Pharmacy and Pharmaceutical Sciences, and; i Glycobiology Research and Training Center, University of California San Diego, La Jolla, California 92093,; the b School of Chemistry and Molecular Biosciences and; c Australian Infectious Diseases Research Centre, The University of Queensland, St. Lucia, Queensland 4072, Australia,; the d Department of Oral and Molecular Microbiology, Osaka University Graduate School of Dentistry, Suita, Osaka 565-0871, Japan,; the f Department of Microbiology and Immunology, Faculty of Pharmacy, Cairo University, Cairo 11562, Egypt,; the g Department of Pediatrics, Laboratory of Pediatric Infectious Diseases, Radboud University Medical Centre, 6500 HC Nijmegen, The Netherlands,; the j Department of Mathematics and Statistics, San Diego State University, San Diego, California 92182,; the k Queensland Health Forensic and Scientific Services, Coopers Plains, Queensland 4108, Australia,; the l Queensland Children's Medical Research Institute, Herston, Queensland 4029, Australia, and; the n Rady Children's Hospital, San Diego, California 92123

**Keywords:** Bacterial Pathogenesis, Hyaluronan, Hyaluronate, Infectious Disease, Streptococcus Pyogenes (S. Pyogenes), Group A Streptococcus, Hyaluronate Lyase, Hyaluronic acid Capsule, Invasive Disease, Nonencapsulated

## Abstract

A recent analysis of group A *Streptococcus* (GAS) invasive infections in Australia has shown a predominance of M4 GAS, a serotype recently reported to lack the antiphagocytic hyaluronic acid (HA) capsule. Here, we use molecular genetics and bioinformatics techniques to characterize 17 clinical M4 isolates associated with invasive disease in children during this recent epidemiology. All M4 isolates lacked HA capsule, and whole genome sequence analysis of two isolates revealed the complete absence of the *hasABC* capsule biosynthesis operon. Conversely, M4 isolates possess a functional HA-degrading hyaluronate lyase (HylA) enzyme that is rendered nonfunctional in other GAS through a point mutation. Transformation with a plasmid expressing *hasABC* restored partial encapsulation in wild-type (WT) M4 GAS, and full encapsulation in an isogenic M4 mutant lacking HylA. However, partial encapsulation reduced binding to human complement regulatory protein C4BP, did not enhance survival in whole human blood, and did not increase virulence of WT M4 GAS in a mouse model of systemic infection. Bioinformatics analysis found no *hasABC* homologs in closely related species, suggesting that this operon was a recent acquisition. These data showcase a mutually exclusive interaction of HA capsule and active HylA among strains of this leading human pathogen.

## Teaser

**Background:** Serotype M4 group A *Streptococcus* lack hyaluronic acid (HA) capsule, but are capable of causing human disease.

**Results:** Encapsulation was achieved by introducing the *hasABC* capsule synthesis operon in the absence of HA-degrading enzyme hyaluronate lyase (HylA).

**Conclusion:** Capsule expression does not enhance M4 GAS virulence.

**Significance:** We demonstrate a mutually exclusive interaction between GAS capsule and HylA expression.

## Introduction

The Gram-positive bacterium *Streptococcus pyogenes*, commonly known as group A *Streptococcus* (GAS),^3^ is a human-specific pathogen ranked among the top 10 etiological agents of infection-related deaths worldwide (1). Annually, GAS is responsible for ∼700 million cases of superficial throat (pharyngitis) and skin (impetigo) infections and ∼650,000 cases of potentially fatal severe invasive infections (*e.g.* bacteremia/sepsis, necrotizing fasciitis, and streptococcal toxic shock syndrome), with an attendant mortality rate of ∼25% (1). GAS strains are distinguished serologically on the basis of the immunovariable M protein (2), a major surface-anchored virulence factor that promotes resistance to opsonophagocytosis (3). Throughout much of the world, M1 is the most frequently isolated serotype from GAS infections, followed by serotypes M12, M28, M3, and M4 (4). A key factor in the resurgence of severe invasive GAS infections over the past 30 years has been the global dissemination of a hypervirulent clone belonging to the M1T1 serotype (5).

The surface capsule of GAS is composed solely of hyaluronan or hyaluronic acid (HA), a high molecular mass polymer of alternating glucuronic acid and *N*-acetylglucosamine residues. The GAS capsule is structurally identical to the HA widely distributed throughout human tissues, allowing GAS to mimic host structures and thwart detection by the host immune system (5). The capsule promotes GAS survival by obstructing antibody binding to epitopes on the bacterial surface, complement deposition (6), and opsonophagocytosis (6, 7). Capsular HA contributes to mouse pharyngeal colonization (8), and interacts with CD44 on human keratinocytes to enhance adherence to pharyngeal epithelial cells (9). Nonencapsulated GAS mutants have significantly reduced survival in human blood and are less virulent than encapsulated WT strains in mouse models of invasive GAS infection (10–13), and a nonhuman primate model of pharyngeal colonization (14).

HA capsule biosynthesis is coordinated by the highly conserved *hasABC* synthase operon (15). The *hasA* gene is essential for HA biosynthesis and encodes for hyaluronate synthase, a membrane-bound enzyme that forms the linear HA polymer by the alternate addition of glucuronic acid and β1,3-linked *N*-acetylglucosamine residues (16, 17). Capsule expression is strongly up-regulated upon exposure of GAS to whole human blood (18), and mucoid or highly encapsulated GAS isolates are often associated with pharyngeal persistence, acute rheumatic fever, and severe invasive human diseases (19). Spontaneously arising and irreversible mutations in the control of virulence regulatory system (*covRS*), a two-component regulator that coordinates the expression of ∼10–15% of genes in the GAS genome (20), have been implicated in the initiation and progression of GAS invasive disease (5, 21, 22). Mutations in *covRS* up-regulate HA capsule biosynthesis and a multitude of virulence factors important for neutrophil resistance (21). Consequently, *covRS* mutants display enhanced virulence in mouse models of systemic GAS infection (22). In addition, *covRS* mutation abrogates expression of the broad spectrum cysteine protease streptococcal pyrogenic exotoxin B (SpeB) (21), allowing the accumulation of human plasmin activity on the GAS surface (23). Plasminogen, a glycoprotein circulating in human blood, is the inactive form of plasmin, a broad spectrum serine protease capable of dissolving blood clots and promoting tissue remodeling (24). Streptokinase is a plasminogen-activating protein secreted by most GAS isolates that is highly specific for human plasminogen (25). GAS bind plasmin(ogen) directly through cell surface receptors, including 1) streptococcal surface enolase (α-enolase/SEN) (26); 2) streptococcal surface dehydrogenase (SDH), also known as glyceraldehyde-3-phosphate dehydrogenase (GAPDH) and plasmin receptor (27); 3) plasminogen-binding M-like protein (28); and 4) plasminogen-binding M-like protein-related protein (29). Indirect plasminogen binding may occur through the formation of a streptokinase-plasminogen-fibrinogen (Fg) trimolecular complex that attaches to Fg or plasminogen receptors on the GAS cell surface (30). Sequestered plasmin activity on the GAS cell surface cannot be inhibited by host regulators α2-antiplasmin and α2-macroglobulin (31), allowing GAS to degrade tissue barriers and spread systemically to normally sterile sites (5, 22, 23).

Although it was long assumed that the HA capsule was an essential virulence factor of the pathogen, genomic analysis recently revealed that disease-associated M4 serotype GAS lack the *hasABC* operon (32), are nonencapsulated, yet nevertheless, can replicate in human blood *ex vivo* (33). During recent epidemiology of severe invasive GAS infections in Australian children, M4 GAS surpassed M1 as the serotype most frequently isolated from normally sterile sites (34). Here, we utilize molecular genetics and bioinformatics to investigate the pathogenicity of 17 M4 clinical isolates from this emerging epidemiological trend. Three pulsed-field gel electrophoresis (PFGE) patterns and 2 multilocus sequence types (MLST) were identified, with more than 50% of isolates harboring mutations within *covRS*, a characteristic of hyperinvasive GAS. All M4 isolates were nonencapsulated and whole genome sequencing of 2 M4 isolates revealed the complete absence of the *hasABC* capsule biosynthesis operon. We identify and functionally demonstrate a mutually exclusive interaction between GAS HA capsule expression (most serotypes) and expression of a secreted hyaluronate lyase (HylA) (35), which is functional in M4 GAS but harbors an inactivating mutation in encapsulated strains. The implications of this dynamic upon GAS invasive disease pathogenesis and evolution are considered in light of these new observations.

## EXPERIMENTAL PROCEDURES

### 

#### 

##### Bacterial Strains and Growth Conditions

M4 GAS strains were isolated from children aged 1–14 years hospitalized with severe invasive infections in Queensland, Australia, between February 2001 and May 2009 (Table 1) (34). M1T1 GAS strain 5448 was isolated from a patient with toxic shock syndrome and necrotizing fasciitis (36). The highly invasive animal passaged variant, 5448AP, is a hyperencapsulated *covS* mutant (22). Isogenic nonencapsulated mutant 5448Δ*hasA* was described previously (37). GAS strain 4063-05 (*emm*4, T-type 4) was isolated in 2005 from the blood of a patient in Georgia, USA. GAS was propagated at 37 °C on Todd-Hewitt agar, or in static liquid cultures of Todd-Hewitt broth (THB, Hardy Diagnostics). When necessary, the growth medium was supplemented with 5 μg/ml of erythromycin or 2 μg/ml of chloramphenicol.

##### Sequence Typing and PFGE

*emm* sequence typing was undertaken using established criteria from the Centers for Disease Control and Prevention. T-typing was performed essentially as described elsewhere (38). MLST was undertaken using the primers listed at the Centers for Disease Control and Prevention and the PCR conditions described at the *S. pyogenes* MLST database. Genomic DNA digests were compared by PFGE using the CHEF-DR II System (Bio-Rad) as described previously (39).

##### HA Capsule Assays

Capsular HA was extracted according to the method of Hollands *et al*. (37). Bacterial cultures were grown to mid-log phase (*A*_600_ = 0.4) in THB and serially diluted for colony-forming unit (cfu) enumeration. 5 ml of culture was centrifuged and resuspended in 500 μl of sterile Milli-Q water. 400 μl of bacterial suspension was added to 1 ml of chloroform, shaken for 5 min in a Mini-BeadBeater-8 (Biospec Products), and clarified by centrifugation at 13,000 × *g* for 10 min. HA in the aqueous phase was quantified using the ELISA HA Test Kit (Corgenix), as per the manufacturer's directions.

##### Glycan Analysis

HA was purified from the aqueous phase by DEAE-Sephacel chromatography and analyzed by high-performance anion-exchange chromatography with pulsed amperometric detection (HPAEC-PAD) for monosaccharides representing HA. Briefly, HA present in the aqueous phase was loaded on the DEAE column and washed with 5 ml of 50 mm NaOAc, 150 mm NaCl solution (pH 6.0) to remove contaminating protein. DEAE-bound HA was eluted with 1 ml of 50 mm NaOAc, 1 m NaCl (pH 6.0) solution. High salt was removed by desalting the sample over a PD10 cartridge (GE Healthcare). Finally, the sample was lyophilized and used for monosaccharide analysis. HA was hydrolyzed to monosaccharide constituents using 2 n trifluoroacetic acid (TFA) at 100 °C for 6 h. TFA was removed by dry nitrogen flush followed by two times co-evaporation with 50% isopropyl alcohol to ensure complete removal of acid. Finally the sample was dissolved in water and monosaccharide profiling was done on the Dionex ICS-3000 using the CarboPac PA1 column (4 × 250 mm; Dionex). NaOH/NaOAc buffer gradient was used as eluent and the monosaccharides were compared and quantified using known amounts of authentic standards as external calibrants.

##### Multiplex PCR Screening

A conserved 561-bp region of *hasA* was amplified with primers hasA-F1 (5′-aatacaattaattgaagagtatgtaaatagaga-3′) and hasA-R1 (5′-attttgttgctttaaataactttttaattggaa-3′). The conserved *speB* gene was amplified with speB-F (5′-ggggatccagattattaagtcttttagcattaggtgga-3′) and speB-R (5′-gggtcgacctaaggtttgatgcctacaacagcactttg-3′). Platinum PCR SuperMix (Invitrogen) was used with the following temperature-cycling parameters: 95 °C for 2 min; 30 cycles of 95 °C for 30 s, 55 °C for 30 s, 72 °C for 1 min 30 s; 72 °C for 10 min; and 4 °C hold.

##### Whole Genome Sequence Analysis

Genomic fragment libraries were prepared at the Australian Genome Research Facility with the Illumina TruSeq DNA library preparation protocol (40). Random subsets of 1 million read pairs were selected to perform read mapping and *de novo* assembly for comparative analysis against the published M4 GAS strain MGAS10750 (RefSeq accession number NC_008024) (32).

##### SpeB Assays and Western Blots

SpeB protease activity in cell-free stationary phase GAS supernatants was determined using the azocaseinoytic assay (41). Western blot analysis of stationary phase supernatants was performed as previously described using rabbit anti-SpeB IgG (Toxin Technology, Sarasota, FL) (42).

##### covRS and ropB Sequencing

*covRS* PCR products generated using primers P1 and P12 were sequenced by Genewiz (La Jolla, CA) with primers P1–P12 (22). Sequence analysis of *ropB* was performed as described previously using primers RopB-F1–RopB-R17 (43). Sequences were aligned to the *covRS* or *ropB* sequences from M4 GAS strain MGAS10750 (32) using MacVector 11.0.4 software.

##### Expression and Purification of Recombinant HylA Proteins

The *hylA* gene, excluding the N-terminal signal peptide and C-terminal cell wall anchor motif, was PCR amplified from M4 GAS strain 4063-05 and M1T1 GAS strain 5448 using primers pQE30-M4-hylAF (5′-caccatcaccatcacgatacactgacttcaaattcaaaac-3′), pQE30-M4-hylAR (5′-caagctcagctaattccagtacgcggtaagcgtcgttgtc-3′), pQE30-M1-hylAF (5′-caccatcaccatcacgatacactgacttcaaattcagaac-3′), and pQE30-M1-hylAR (5′-caagctcagctaattttattgttgattttgcctgacagga-3′). His_6_ tag expression pQE-30 vector (Qiagen) was linearized by PCR amplification with primers pQE30-M4-hylAvF (5′-cttaccgcgtactggaattagctgagcttggactcctgtt-3′), pQE30-M4-hylAvR (5′-tgaagtcagtgtatcgtgatggtgatggtgatgcgatcct-3′), pQE30-M1-hylAvF (5′-caaaatcaacaataaaattagctgagcttggactcctgtt-3′), and pQE30-M1-hylAvR (5′-tgaagtcagtgtatcgtgatggtgatggtgatgcgatcct-3′). Purified PCR products were assembled using GeneArt Seamless Cloning (Invitrogen). Recombinant His_6_-tagged HylA proteins were expressed and purified using TALON Metal Affinity Resin (Clontech), according to the manufacturer's instructions.

##### Enzymatic Assays

Glycosidase activity assays were performed essentially as previously described (44). HA sodium salt from rooster comb, chondroitin sulfate sodium salt from shark cartilage, heparan sulfate sodium salt from bovine kidney, and chondroitin sulfate B (also known as dermatan sulfate) sodium salt were purchased from Sigma. The substrates were dissolved in 50 mm ammonium acetate buffer (pH 6.5), 10 mm calcium chloride (45). Recombinant HylA (500 or 5,000 pm) and 0.05–0.30 or 1.0 mg/ml substrates were incubated in the ammonium acetate buffer at 37 °C. The rate of substrate degradation was measured by monitoring the increase of *A*_232_ over time. The kinetic parameters of M4 HylA with concentration ranges of 0.05–0.30 mg/ml of HA at 37 °C were calculated using the following formula and Lineweaver-Burk double-reciprocal plots,

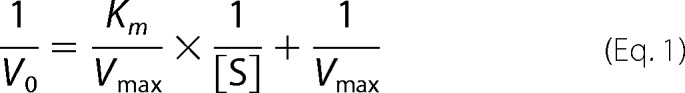

*V*_0_ is the initial reaction rates (*A*_232_/min), *K_m_* is the Michaelis-Menten constant, *V*_max_ is the maximum reaction velocity, and [S] is the substrate concentration.

##### Bioinformatic Analysis

The distribution of the HylA-encoding gene, *hylA*, was examined essentially as previously described (16). Briefly, the SEED and NCBI RefSeq genomic databases were searched for HylA protein homologs using BLASTP and subsystem analysis for protein similarity. When no annotated protein homologs were found in a genome, the absence of *hylA* was confirmed by the lack of tBLASTN matches.

##### Construction of ΔhylA Mutants

Allelic exchange mutagenesis was performed as previously described (16) using primers hylA-XhoI-upF (5′-cggctcgagcacgcgagcacgaacagacttcac-3′), hylA-upR-cat (5′-ccagtgatttttttctccattgataaattcctccaatataaaaatgagataataaaag-3′), hylA-downF-cat (5′-tggcagggcggggcgtaaaagcttgctgatcaaggaattgcagctaaaaacaatgctc-3′), and hylA-XbaI-downR (5′-cgctctagacgaagcagctactattatggaatctg-3′). The precise in-frame allelic exchange of *hylA* with the chloramphenicol resistance gene (*cat*) in 5448Δ*hylA* and 4063-05Δ*hylA* was verified by PCR and HylA activity assays (46).

##### Complementation of ΔhylA mutants

The *hylA* genes from GAS strains 4063-05 (serotype M4) and 5448 (serotype M1) were PCR amplified using forward primer M1-HylA-For-EcoRI (5′-agcgaattcgtgaatacttatttttgcacac-3′) or M4-HylA-For-NsiI (5′-agcatgcatctaaatccttaagtctttcttac-3′), and reverse primer HylA-Rev-BamHI (5′-agcggatccttattgttgattttgcctgac-3′). PCR products were cloned into the erythromycin-resistant plasmid pDCerm to create pHylA (expressing active M4 GAS HylA) and pHylA* (expressing inactive M1 GAS HylA). The plasmids were electroporated into 5448Δ*hylA* to construct complemented strains 5448Δ*hylA* pHylA and 5448Δ*hylA* pHylA*.

##### Capsule Expression in M4 GAS

The *hasABC* operon from M1 GAS strain 5448 was PCR amplified using primers hasABC-F-XbaI (5′-ggtctagagtgcctatttttaaaaaaactttaat-3′) and hasABC-R-BamHI (5′-ggggatccttactttgaatgtgttggtactttac-3′) and cloned into pDCerm. The resultant plasmid, pHasABC, was electroporated into WT 4063-05 and 4063-05Δ*hylA* to construct 4063-05 pHasABC and 4063-05Δ*hylA* pHasABC, respectively. Capsule expression was quantified using the HA test kit as described above.

##### Whole Blood Survival

Bacterial survival post 2 h incubation in whole human blood was analyzed as described previously (47).

##### C4BP Pull-down and Adherence Assays

Recombinant His_6_-tagged M proteins and the C4BP fragment (C4BPα1–2) (48) were expressed and purified as previously described (37, 38). C4BP pull-down assays were performed by mixing 10 μg of C4BPα1–2 and 20 μg of M protein in 50 μl of binding buffer (300 mm NaCl, 50 mm sodium phosphate buffer, pH 8.0, 50 mm imidazole, 0.1% (v/v) Triton X-100) at 37 °C for 30 min. 20 μl of Ni^2+^-nitrilotriacetic acid-agarose beads (Qiagen) equilibrated in binding buffer were added to the protein mixture and incubated for 30 min at 37 °C under agitation. The beads were washed three times with 200 μl of binding buffer and proteins were eluted by boiling for 5 min in non-reducing 5× SDS-PAGE sample buffer. Fractions corresponding to unbound and bound proteins were resolved by non-reducing SDS-PAGE and visualized with Coomassie stain. Microtiter plate adherence assays were conducted according to the method of Dahesh *et al.* (47) with 4 μg of purified human C4BP (Complement Technology, Tyler, TX).

##### Fibrinogen Binding

Mid-log phase GAS (*A*_600_ = 0.4) in 10-ml culture volumes was centrifuged, resuspended in 5 ml of PBS, and 100-μl aliquots were added to a round bottom 96-well plate (Costar). Human Fg conjugated with Alexa Fluor 488 (Molecular Probes) was added to a final concentration of 100 μg/ml and the plate incubated at 37 °C for 1 h with shaking. The plate was centrifuged at 500 × *g* for 10 min and wells were washed 3 times with 200 μl of PBS. Bacteria were resuspended in 150 μl of PBS and analyzed by flow cytometry. The average geometric mean of samples without fibrinogen was subtracted from each strain to adjust for background.

##### Cell Surface Plasmin Activity

GAS cultures were grown overnight to stationary phase. The next day, 300 μl of culture was added to 3 ml of THB supplemented with 1 unit/ml of human plasminogen (Calbiochem) and 7 μm human fibrinogen (Calbiochem) to facilitate cell surface plasmin acquisition, or THB only as the negative control. Cultures were grown to *A*_600_ = 0.4, divided into 3 × 1-ml aliquots in siliconized tubes, centrifuged for 5 min at 6,000 × *g*, and bacterial pellets were washed once with 1 ml of sterile PBS. Following resuspension in 200 μl of PBS, 10-μl aliquots were collected for cfu enumeration, prior to transferring 180 μl into V-bottom 96-well plates (Costar) and adding 20 μl of substrate S-2251 (Chromogenix). The plate was incubated in the dark for 1 h at 37 °C, centrifuged for 10 min at 500 × *g*, and 100 μl from each well was transferred into a flat bottom 96-well plate (Costar). The *A*_405_ was measured using SpectraMax 250 (Molecular Devices). Cell surface plasmin activity was calculated as absorbance units/cfu. The following control wells were used: positive control, 1 unit/ml of human plasminogen + 1 μg of streptokinase from group C *Streptococcus* (Sigma); negative control, 1 unit/ml of human plasminogen only; substrate negative control, PBS only.

##### Neutrophil Killing Assays

Human neutrophils were isolated from venous blood using the PolymorphPrep system (Axis-Shield) and resuspended to 2 × 10^5^ cells/100 μl in RPMI 1640 + 2% FBS heat inactivated for 30 min at 56 °C. Survival assays were performed as previously described (47). Briefly, 100 μl of neutrophil suspension was seeded into 96-well plates and 100 μl of mid-log phase bacteria in RPMI + 2% heat inactivated FBS were added for a multiplicity of infection of 1 (M4 GAS) or 0.1 (M1 GAS). The assay plate was centrifuged at 500 × *g* for 10 min and incubated for 15 min at 37 °C + 5% CO_2_. Aliquots were serially diluted and plated onto Todd-Hewitt agar for enumeration. Percent survival was calculated using bacterial control wells grown under the same conditions without neutrophils.

##### Systemic Infection Model

Cohorts of 10-week-old female CD-1 mice (Charles River Laboratories) were inoculated intraperitoneally with ∼10^8^ cfu in 200 μl of PBS, 5% porcine gastric mucin (Sigma), and survival was monitored twice daily for 14 days.

##### Statistical Analysis

Capsular expression levels, SpeB protease activity, whole blood survival, C4BP binding, fibrinogen binding, and plasmin activity assays were compared by one-way analysis of variance. Neutrophil survival was analyzed using the Student's *t* test. Kaplan-Meier survival curves were compared using the log-rank test. Differences were considered significantly different at *p* < 0.05. All statistical analyses were performed with GraphPad Prism version 5.0b (GraphPad Inc.).

##### Ethics Approval

Permission to collect human blood under informed consent was approved by the University of California San Diego (UCSD) Human Research Protection Program. Procedures used for all animal experiments were approved by the UCSD Institutional Animal Care and Use Committee.

## RESULTS

### 

#### 

##### Typing and Genotypic Analysis Suggest That the M4 GAS Isolates Are Not Clonal

**FIGURE 1.**
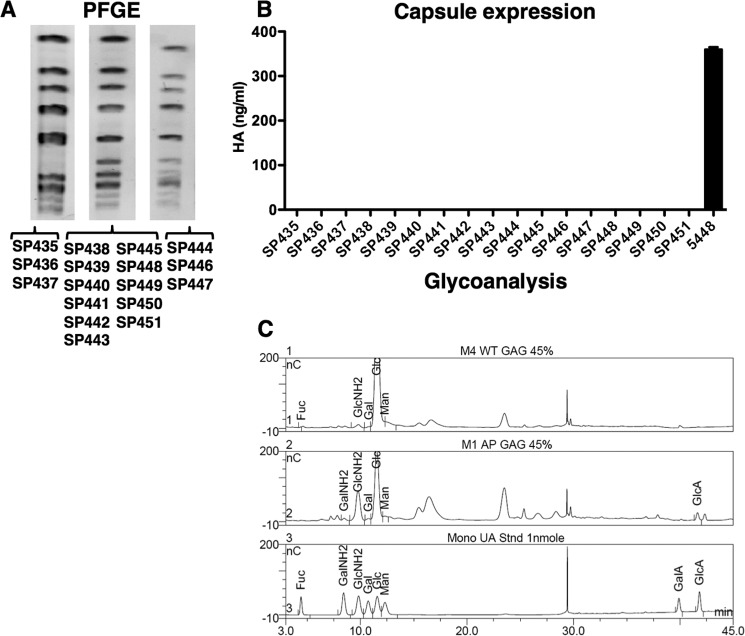
*A,* PFGE typing of 17 clinical M4 GAS isolates associated with invasive disease. Data are representative of 2 independent experiments. *B,* mid-log phase capsule expression levels for M4 strains SP435–SP451 and M1 strain 5448. Values denote arithmetic mean ± S.E. Data were pooled and normalized to 5448 from 2 independent experiments, each performed in triplicate. *C,* monosaccharide composition analysis of hydrolyzed glycosaminoglycan (*GAG*)-enriched fractions from WT M4 GAS, encapsulated WT M1 GAS control strain 5448AP (22), and 1 nmol standards. Abbreviations used are: *Fuc*, fucose; *GalNH*_2_, galactosamine; *GlcNH*_2_, glucosamine; *Gal*, galactose; *Glc*, glucose; *Man*, mannose; *GalA*, galacturonic acid; *GlcA*, glucuronic acid.

Over the past few decades, M1 GAS has been the most frequently isolated serotype from human infections worldwide (4) and the leading cause of life-threatening invasive syndromes (5). However, serotype M4 was the principal serotype associated with a recent report of severe invasive infections in Queensland, Australia, accounting for 16% of isolates compared with 8% for M1 (34). 17 such M4 isolates from this region, designated SP435–SP451, were obtained from children aged 1 to 14 years with invasive GAS infections between 2001 and 2009 (Table 1). SP435 and SP436 were highly virulent strains isolated from brothers hospitalized for 2–3 weeks (supplemental Table S1). The worldwide resurgence of severe invasive GAS infections over the past three decades has been attributed to the emergence of a single globally disseminated serotype M1T1 GAS clone (20). To determine whether the M4 isolates were clonal in origin, genomic DNA extracts were analyzed by PFGE. Three distinct PFGE patterns were identified, with the majority (65%) of M4 isolates sharing the same pattern (Fig. 1*A*). MLST classified the strains into 2 groups, with 15 of 17 (88%) identified as ST 39 (Table 1). SP449 and SP451 share a unique and hitherto unidentified *mutS* allele (supplemental Table S2), and have yet to be assigned a ST by the *S. pyogenes* MLST database.

**TABLE 1:** **Clinical origin of serotype M4 GAS isolates** The table uses the following abbreviations: F, female; M, male; ST, multilocus sequence type; tba, newly identified MLST to be assigned by *S. pyogenes* MLST database curators; UTI, urinary tract infection.

GAS isolate	Date*^a^*	DOB*^b^*	Sex	Tissue*^c^*	*emm* type	*emm* subtype	*tee* type	ST	Physician notes
SP435*^d^*	20 Feb-01	07 Jul-99	M	Blood	4	4.15	4	39	Septic arthritis *E. coli* UTI
SP436*^d^*	20 Feb-01	11 Feb-97	M	Blood	4	4.15	4	39	Community acquired sepsis, with knee pain
SP437	24 Jun-02	21 Sep-00	M	Blood	4	4.15	4	39	NA*^e^*
SP438	06 Jul-06	22 Mar-04	M	Blood	4	4.0	4	39	Nonspecific rash, pedal oedema
SP439	21 Aug-06	13 Apr-04	M	Blood	4	4.0	4	39	Abscess?
SP440	NA	07 Apr-05	M	Blood	4	4.0	4	39	Generally unwell
SP441	11 Apr-07	08 Sep-05	F	Blood	4	4.0	4	39	NA
SP442	10 May-07	25 Jan-00	M	Blood	4	4.0	4	39	Hand-foot-and-mouth disease?
SP443	01 Nov-07	01 May-02	M	Abscess	4	4.0	4	39	NA
SP444	28 Apr-08	19 Feb-06	M	Blood	4	4.0	4	39	NA
SP445	13 May-08	01 Dec-93	M	Blood	4	4.0	4	39	NA
SP446	29 May-08	12 Jan-06	F	Blood	4	4.0	4	39	NA
SP447	05 Sep-08	18 Sep-01	F	Tissue	4	4.0	4	39	Severe sepsis with multiorgan failure
SP448	29 Dec-08	27 Feb-04	M	Blood	4	4.0	4	39	NA
SP449	02 Feb-09	12 Oct-03	M	Blood	4	4.0	4	tba	Vomiting + rashes
SP450	26 May-09	12 Feb-01	M	Blood	4	4.0	4	39	Febrile, seizure
SP451	09 Feb-09	12 Oct-03	M	Throat	4	4.0	4	tba	NA

*^a^* Date isolate received; day, month, and year.

*^b^* Date of birth; day, month, and year.

*^c^* Human tissue from which M4 GAS was isolated.

*^d^* SP435 and SP436 were isolated from brothers; see supplemental Table SI for detailed clinical information.

*^e^* NA, data not available.

##### M4 GAS Are Nonencapsulated and Lack the hasA Gene

A recent study identified capsule-deficient M4 GAS (33). To ascertain whether our geographically distinct M4 GAS isolates were similarly nonencapsulated, mid-logarithmic phase cultures were screened for HA capsule expression levels using a commercial ELISA-based kit. All M4 isolates were negative for capsule expression, compared with M1 GAS positive control strain 5448 (Fig. 1*B*). To corroborate the ELISA data, we undertook monosaccharide composition analysis of hydrolyzed glycosaminoglycan-enriched fractions from WT M4 GAS and encapsulated M1 GAS control strain 5448AP (22). Glucosamine (GlcNH_2_) and glucuronic acid (GlcA), the constituents of HA, were detected for 5448AP (Fig. 1*C*), verifying capsule expression in M1 GAS. The double peak near GlcA is characteristic of HA. In contrast, M4 GAS was completely deficient in GlcA and had very small amounts of GlcNH_2_, compared with 5448AP (Fig. 1*C*). These data confirm that M4 GAS lack HA capsule.

**FIGURE 2.**
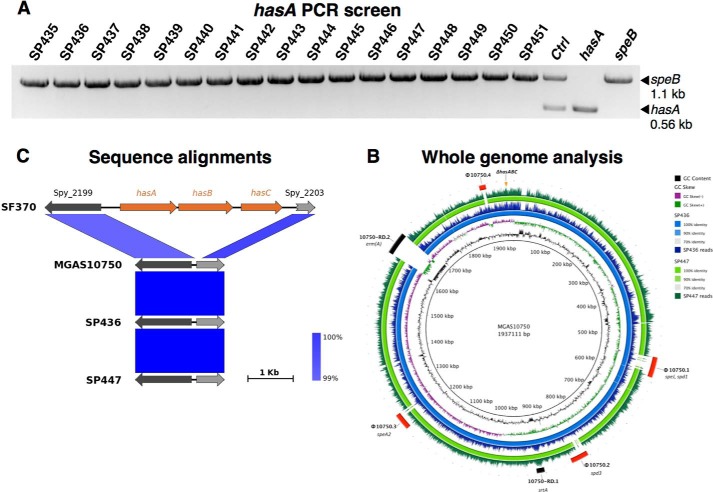
*A,* multiplex PCR screening for *hasA*, the essential gene for capsule biosynthesis, and control gene *speB* encoding cysteine protease SpeB. Data are representative of 2 independent experiments. *B,* genome-wide comparison of SP436 and SP447 to the published M4 GAS genome MGAS10750 (RefSeq accession number NC_008024) (32). A total of 7,761,941 and 8,310,842 read pairs were obtained for SP436 and SP447, respectively, corresponding to an estimated average coverage of 862X and 923X. The draft genomes of SP436 and SP447 consist of 75 and 40 scaffolds, respectively, each concatenated into a single circular chromosome of an estimated size of 1.89 and 1.78 Mbp with G + C contents of 38.25% (SP436) and 38.33% (SP447). The *innermost circles* represent the GC content (*black*) and GC skew (*purple*/*green*) of the central reference strain MGAS10750. The BRIG representation shows for each strain, SP436 (*blue*) and SP447 (*green*), respectively, from the innermost to outermost, the sequence similarity and distribution of the number of reads mapped onto the central reference using a window size of 500. The *outermost circle* represents previously reported regions of difference in MGAS10750, including prophage elements φ10750.1 to φ10750.4 (*red*) and integrative conjugative elements 10750.RD-1 and 10750.RD-2 (*black*). M4 GAS lack the *hasABC* capsule biosynthesis operon, the location of which is depicted on the outermost ring (*orange triangle*). *C,* schematic alignment of the *hasABC* operon and flanking regions for serotype M4 (SP436, SP447, MGAS10750) and M1 (SF370). M4 GAS are deficient in *hasABC* and have conserved flanking regions with M1 GAS (99% sequence identity).

Multiplex PCR screening of purified genomic DNA revealed that none of the M4 isolates contained the essential capsule synthesis gene *hasA* (Fig. 2*A*) (17), consistent with the previous report (33). In contrast, all M4 isolates were positive for the control gene, *speB*, encoding for the ubiquitous cysteine protease SpeB (Fig. 2*A*).

##### M4 GAS Lack the hasABC Capsule Biosynthesis Operon

To validate the absence of *hasA* and further investigate the enhanced virulence potential of the newly emerged M4 GAS, two isolates with different PFGE patterns, SP436 and SP447, were subjected to whole genome sequence analysis (Fig. 2*B*). Comparison of SP436 and SP447 genomic content to the sequenced M4 genome MGAS10750 (RefSeq accession number NC_008024) (32) reveals >99% identity at the nucleotide level with most of the sequence divergence between the strains confined to mobile genetic elements. Similar to MGAS10750 (33), SP436 and SP447 lack the *hasABC* capsule biosynthesis operon, strongly suggesting that this operon is absent in ancestral M4. The genomic region flanking *hasABC* is highly conserved between M4 GAS strains (SP436, SP447, and MGAS10750) and the M1 reference strain SF370 (RefSeq accession number NC_002737) (49) (Fig. 2*C*).

When compared with MGAS10750, the genomes of SP436 and SP447 harbor the same integrative conjugative element 10750.RD-1 encoding sortase SrtA, but are missing integrative conjugative element 10750.RD-2, which confers resistance to erythromycin (Fig. 2*B*). SP436 contains the same four prophages previously identified for MGAS10750, as well as one additional putative prophage carrying the streptodornase encoding gene *sdn*. In contrast, all four prophages in SP447 have undergone substantial deletion events and become remnants, whereas maintaining all their respective cargo genes intact. Functional annotation of the predicted coding DNA sequences also revealed that many previously identified virulence factors were present in the SP436 and SP447 genomes, including streptolysin O, IgG-degrading enzyme IdeS, streptococcal mitogenic exotoxin Z, SpeB, and C5a peptidase, but not streptococcal inhibitor of complement nor serum opacity factor.

##### The Majority of M4 GAS Isolates Are SpeB-negative covRS Mutants

**FIGURE 3.**
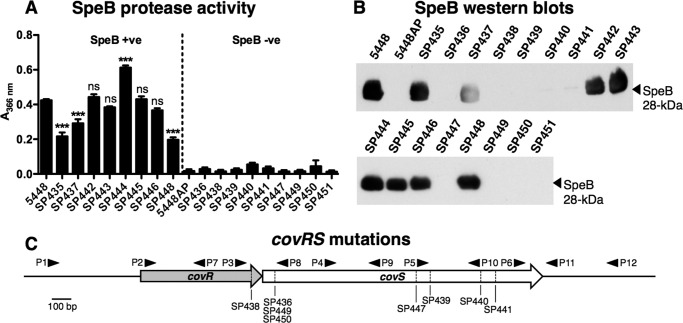
*A,* quantification of SpeB protease activity in stationary phase culture supernatants of M4 GAS (SP435-SP451), M1 GAS positive control (5448), and M1 GAS negative control (5448AP), a SpeB-deficient *covS* mutant (22). Each *bar* denotes the arithmetic mean ± S.E. Data were pooled and normalized to 5448 from 2 independent experiments, each performed in triplicate. ***, *p* < 0.001; ns, no significant difference compared with 5448. *B*, SpeB Western blot analysis of stationary phase culture supernatants. The 28-kDa SpeB protease band is indicated. *C, covRS* DNA sequence analysis of M4 GAS isolates. The positions of the *covRS* mutations and primers used for sequence analysis (*P1–P12*) are indicated.

Mutations within the *covRS* two-component regulatory system have been implicated in the initiation of GAS invasive disease (5, 21). To investigate whether the M4 isolates in this study underwent selection for *covRS* mutation in the human host, we first screened the M4 panel for loss of SpeB protease activity. A significant proportion of M4 isolates, 9 of 17 (53%), were negative for SpeB activity (Fig. 3*A*), suggesting that some may harbor *covRS* mutations. Western blot analysis of stationary phase culture supernatants confirmed that isolates lacking SpeB activity did not secrete an active 28-kDa SpeB protease into the extracellular milieu (Fig. 3*B*). Sequence analysis of SpeB-negative M4 isolates confirmed that 8 of 9 (89%) were *covRS* mutants (Fig. 3*C*), with 3 isolates (SP436, SP449, and SP450) harboring the same *covS* deletion mutation at nucleotide (nt) 77 resulting in a truncated CovS protein (Table 2). SP438 was the only *covR* mutant, containing a Cys to Thr substitution mutation at nt 575 of the *covR* gene. SP451 contained 2 mutations in *ropB*, also known as *rgg*, a transcriptional regulator associated with the loss of SpeB expression in some invasive disease isolates (Table 2) (50). Taken together, these data confirm that several M4 GAS isolates associated with human invasive disease have either *covRS* or *ropB* mutations eliminating SpeB protease activity (5).

**TABLE 2:** **covRS and *ropB* DNA sequence analysis of SpeB-negative M4 GAS isolates from patients with invasive infections**

M4 isolate	Mutation*^a^*	Consequence*^b^*
SP436	Δnt 77 *covS*	Truncation in CovS
SP438	C to T nt 575 *covR*	Thr to Ile aa 192 CovR
SP439	C to T nt 838 *covS*	His to Tyr aa 280 CovS
SP440	C to A nt 1,136 *covS*	Ala to Asp aa 379 CovS
SP441	Δnt 1,215−1,219 *covS*	Truncation in CovS
SP447	G to A nt 780 *covS*	Met to Ile aa 260 CovS
SP449	Δnt 77 *covS*	Truncation in CovS
SP450	Δnt 77 *covS*	Truncation in CovS
SP451	C to T nt 347 *ropB*	Ser to Leu aa 116 RopB
SP451	G to T nt 553 *ropB*	Glu to stop codon aa 185 RopB

*^a^* Mutation positions are based on nucleotide (nt) position in the *covR*, *covS*, or *ropB* genes, relative to each ATG start codon.

*^b^* Substitutions in CovR, CovS, and RopB are based upon amino acid (aa) position in each open reading frame, relative to each start codon.

##### M4 HylA Specifically Degrades HA

**FIGURE 4.**
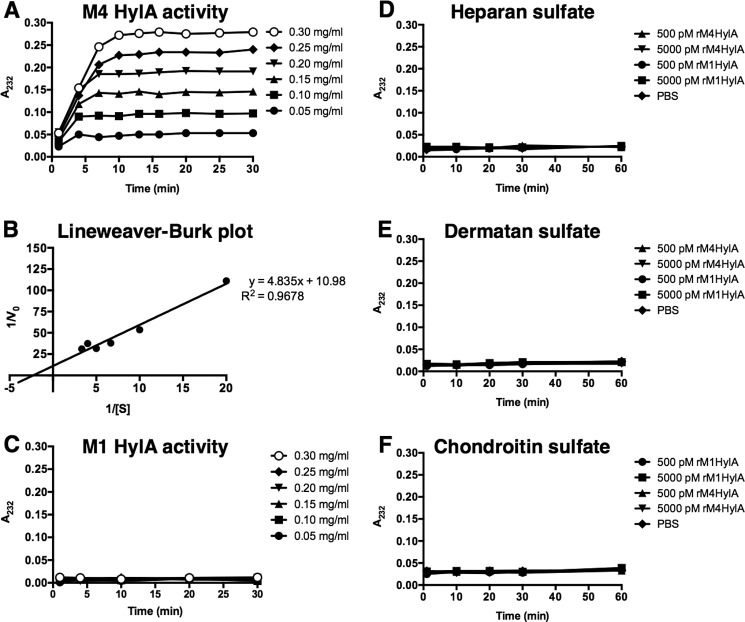
*A,* time-dependent kinetics of recombinant M4 HylA digestion of HA substrate at various concentrations (0.05–0.30 mg/ml). *B*, Lineweaver-Burk double reciprocal plot showing 1/*V*_0_ *versus* 1/[S] (*R*^2^ = 0.9678). *V*_0_ represents initial reaction rate, and [S] represents the HA substrate concentration. *C,* time-dependent kinetics of recombinant M1 HylA digestion of HA substrate at various concentrations (0.05–0.30 mg/ml). *D–F,* time-dependent kinetics of recombinant M4 and M1 HylA (500 or 5,000 pm) digestion of heparan sulfate (1 mg/ml), dermatan sulfate (1 mg/ml), chondroitin sulfate (1 mg/ml), and PBS negative control. Data are representative of 3 independent experiments, each performed in triplicate.

Some Gram-positive bacteria, including *Streptococcus pneumoniae* (pneumococcus), *Streptococcus suis*, and *Staphylococcus aureus* secrete an active HA-degrading HylA enzyme. Yet, in most clinically relevant GAS serotypes, such as M1, this enzyme is inactivated by a single nucleotide substitution resulting in an amino acid change from Asp to Val at position 199 of the lyase (51). The only reported GAS serotypes with a lyase possessing Asp-199 are M4 and M22 (51). To evaluate the enzymatic activity of HylA from M4 and M1 GAS, recombinant His_6_-tagged HylA protein from each serotype was expressed in *Escherichia coli* and purified by TALON affinity chromatography. Recombinant M4 HylA was enzymatically active and degraded HA in a substrate concentration-dependent manner (Fig. 4*A*). The kinetic parameters *K_m_* and *V*_max_ for M4 HylA were 0.440 mg/ml and 0.091, respectively, as estimated from the Lineweaver-Burk double-reciprocal plot (Fig. 4*B*). In contrast, recombinant M1 HylA was enzymatically inactive and unable to digest HA (Fig. 4*C*). M4 HylA was highly specific for HA and did not degrade other glycosaminoglycans, including heparan sulfate (Fig. 4*D*), dermatan sulfate (Fig. 4*E*), and chondroitin sulfate (Fig. 4*F*).

##### The hylA Gene Is Ancestral and hasABC Was Recently Acquired by Some GAS Serotypes

**FIGURE 5.**
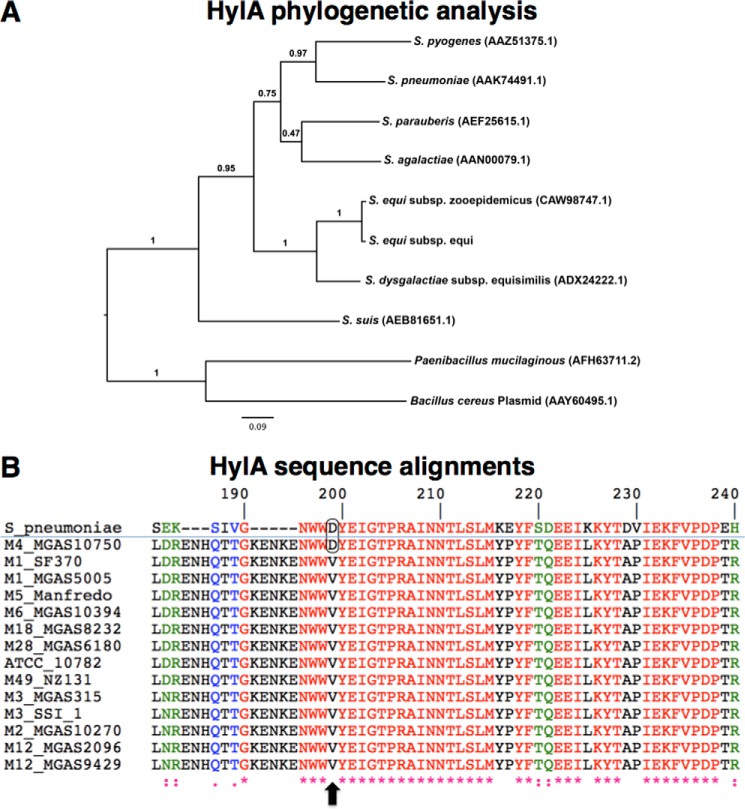
*A,* maximum likelihood phylogenetic tree of HylA proteins in different streptococcal species. Approximate likelihood ratios are shown for branch support. *B,* section of a multiple sequence alignment (using ClustalW) of HylA protein in sequenced GAS strains, with *S. pneumoniae* used as an outgroup homolog, showing a Asp to Val substitution that reportedly abolishes the hyaluronidase activity of HylA (51). Only M4 and M22 GAS serotypes are known to possess an active HylA enzyme (51).

HylA is well conserved in closely related genomes including *Streptococcus agalactiae*, *S. pneumoniae, S. suis,* and *S. aureus* suggesting that *hylA* is unlikely to have been independently acquired by these genomes, but may rather be ancestral among streptococcal species (Fig. 5*A*; supplemental Table S3). We hypothesize that M1 GAS and other encapsulated serotypes acquired *hasABC* more recently than *hylA*, resulting in concurrent HA synthesis and degradation. Preservation of capsule bestows upon GAS resistance to phagocytosis and enhanced survival *in vivo*, which may have provided selection pressure for inactivating mutations in *hylA*. Although current data do not exclude that the *hylA* might be horizontally acquired, the high degree of sequence conservation in HylA proteins among streptococci and other bacterial species (Fig. 5*B*; supplemental Table S3) suggests that *hylA* acquisition may have been ancestral to the branching of streptococci. It is possible that *hylA* is not metabolically essential and that it might be detrimental to certain bacterial products, because a few species have lost this gene (*e.g. Streptococcus mutans*, *Streptococcus uberis*, and *Streptococcus thermophilus*) (supplemental Table S3).

##### High Levels of Capsule Can Be Induced in M4 GAS Isolates Lacking hylA

**FIGURE 6.**
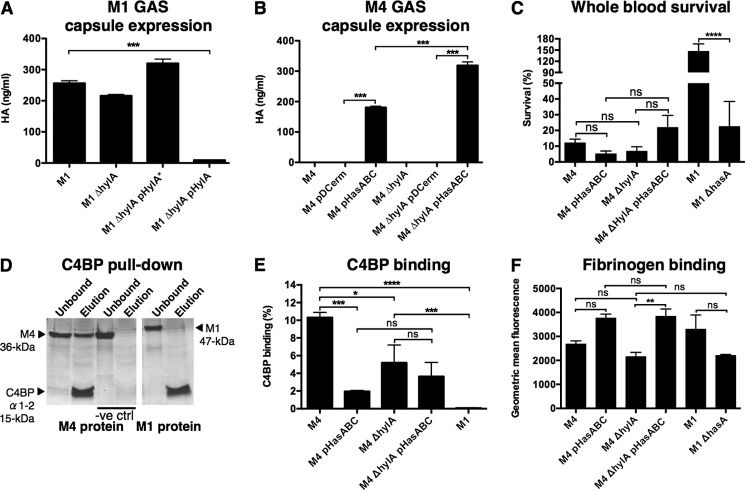
*A,* capsule expression levels of WT clinical M1 GAS isolate 5448 and isogenic Δ*hylA* mutant. The M1 Δ*hylA* mutant was complemented with a plasmid expressing the inactive hyaluronidase (HylA) from M1 GAS (pHylA*), or the active HylA from M4 GAS (pHylA). *B,* capsule expression of WT clinical M4 GAS isolate 4063-05 and isogenic Δ*hylA* mutant. M4 WT and Δ*hylA* were transformed with a plasmid expressing the *hasABC* capsule synthesis operon (pHasABC) or empty vector (pDCerm). *C,* whole blood survival of nonencapsulated WT M4, encapsulated M4 (M4 pHasABC), nonencapsulated *hylA* mutant (M4 Δ*hylA*), encapsulated *hylA* mutant (M4 Δ*hylA* pHasABC), encapsulated M1 GAS, and M1 GAS acapsular control (M1 Δ*hasA*) following a 2-h incubation in whole human blood *ex vivo. D,* association of His_6_-tagged C4BPα1–2 with M4 protein in co-precipitation (pull-down) assays. C4BPα1–2 was mixed with M protein in binding buffer for 30 min at 37 °C. Ni^2+^-nitrilotriacetic acid-agarose beads were added and incubated for 30 min at 37 °C. The beads were washed with binding buffer to remove unbound protein. Bound protein was eluted by boiling in non-reducing sample buffer. Fractions corresponding to unbound and bound protein were resolved by non-reducing SDS-PAGE and visualized with Coomassie stain. *E*, C4BP binding of nonencapsulated M4 GAS (WT and Δ*hylA*), encapsulated M4 GAS (M4 pHasABC and Δ*hylA* pHasABC), and encapsulated WT M1 GAS. *F,* fibrinogen binding of nonencapsulated M4 GAS (WT and Δ*hylA*), encapsulated M4 GAS (M4 pHasABC and M4 Δ*hylA* pHasABC), encapsulated WT M1 GAS, and nonencapsulated M1 GAS (M1 Δ*hasA*). All values denote arithmetic mean ± S.E. Data were pooled and normalized from 2 independent experiments, each performed in triplicate. *, *p* < 0.05; **, *p* < 0.01; ***, *p* < 0.001; ****, *p* < 0.0001; *ns*, not significantly different.

To assess whether an active HylA would have the capacity to digest the capsule of the bacterium, we used precise allelic exchange mutagenesis to delete the *hylA* gene in M1 GAS strain 5448 (encoding an inactive HylA). Complementation of M1 Δ*hylA* with a plasmid expressing active HylA from M4 GAS (pHylA), but not the inactive HylA from M1 GAS (pHylA*), completely abolished capsule expression (Fig. 6*A*). Conversely, to determine whether M4 GAS is capable of synthesizing capsule in the absence of HylA, we constructed a *hylA* allelic exchange mutant in M4 GAS strain 4063-05, a human blood isolate. Transformation of M4 Δ*hylA* with pHasABC, a plasmid expressing the *hasABC* operon from M1 GAS, resulted in capsule expression (Fig. 6*B*). However, the amount of capsule detected for WT M4 GAS transformed with pHasABC (M4 pHasABC) was significantly less, compared with M4 Δ*hylA* pHasABC (Fig. 6*B*). As a corollary, these findings suggest that HylA inactivation prevents capsule degradation in GAS serotypes containing the *hasABC* operon.

##### Capsule Expression in M4 GAS Does Not Enhance Whole Blood Survival, Reduces C4BP Binding, and Has No Effect on Fibrinogen Binding

Encapsulated M4 GAS (M4 pHasABC) did not display enhanced survival in whole human blood *ex vivo* compared with the nonencapsulated WT M4 strain (Fig. 6*C*). In contrast, whole blood survival for WT M1 GAS was superior to the acapsular M1 Δ*hasA* mutant (Fig. 6*C*), consistent with previous reports (41). Several human pathogens, including *S. aureus* (52), *S. pneumoniae* (53), *S. agalactiae* (group B *Streptococcus*) (54), *Neisseria gonorrhoeae* (55), and certain GAS serotypes, including M4 (56), bind human complement regulatory protein C4BP to prevent complement deposition and activation on the bacterial cell surface (57). GAS C4BP binding can be mediated by certain M proteins, including M4 protein (Fig. 6*D* and (56)), but not M1 protein (Fig. 6*D* and Ref. 58). Next, we assessed whether capsule expression in M4 GAS affects the binding of purified human C4BP to the bacterial surface. In comparison to nonencapsulated WT M4 GAS, ectopic capsule expression in M4 pHasABC significantly reduced C4BP binding (Fig. 6*E*). M4 Δ*hylA* bound less C4BP than M4 WT (Fig. 6*E*), suggesting a role for HylA in M4 GAS C4BP binding. Capsule synthesis in M4 Δ*hylA* pHasABC exhibited a trend toward reduced C4BP binding compared with M4 Δ*hylA*; however, this difference did not reach statistical significance (Fig. 6*E*).

Human Fg is a plasma glycoprotein involved in the blood coagulation cascade and wound healing processes (59). Fg binding by GAS enhances resistance to phagocytosis by preventing complement C3 convertase deposition on the bacterial surface (60, 61), and forms a proinflammatory supramolecular network with M protein that activates neutrophils and contributes to the pathophysiology of streptococcal toxic shock syndrome (62). Capsule deficiency in M4 GAS may enhance Fg binding by fully exposing Fg adhesins on the bacterial surface. To test this hypothesis, the binding of Alexa Fluor 488-labeled human Fg to whole bacteria was assessed by flow cytometry. Nonencapsulated M4 WT, M4 Δ*hylA*, and M1 Δ*hasA* bound equivalent quantities of Fg (Fig. 6*F*). Capsule biosynthesis in M4 Δ*hylA*, but neither M4 nor M1 WT strains, enhanced Fg binding (Fig. 6*F*).

##### Capsule Enhances M4 GAS Plasmin Activity and Neutrophil Survival, but Has No Effect on in Vivo Virulence

**FIGURE 7.**
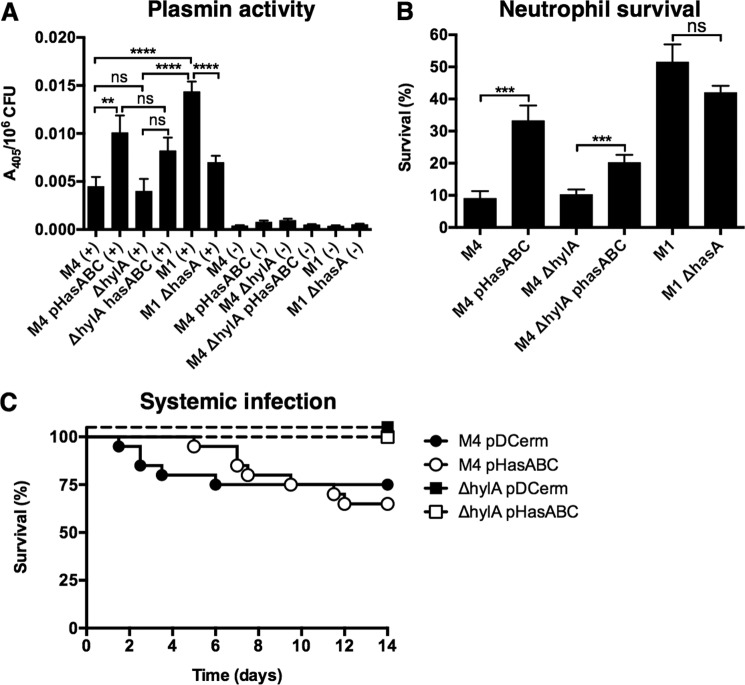
*A*, cell surface plasmin activity of nonencapsulated M4 GAS (WT and Δ*hylA*), encapsulated M4 GAS (M4 pHasABC and M4 Δ*hylA* pHasABC), encapsulated WT M1 GAS, and nonencapsulated M1 GAS (M1 Δ*hasA*). Values denote arithmetic mean ± S.E. Data were pooled and normalized to M1 from 2 independent experiments, each performed in triplicate; *, *p* < 0.05; ***, *p* < 0.001; *ns*, not significantly different. *Plus sign* (+) indicates cultures grown in the presence of human plasminogen; *minus sign* (−), indicates cultures grown in the absence of human plasminogen. *B,* bacterial survival of nonencapsulated M4 GAS (WT and Δ*hylA*), encapsulated M4 GAS (M4 pHasABC and M4 Δ*hylA* pHasABC), encapsulated WT M1 GAS, and nonencapsulated M1 GAS (M1 Δ*hasA*) following exposure to human neutrophils. Values denote arithmetic mean ± S.E. Data were pooled and normalized to M1 from 2 independent experiments, each performed in triplicate. ***, *p* < 0.001; *ns*, not significantly different. *C,* Kaplan-Meier survival curves for nonencapsulated M4 GAS (M4 pDCerm, *n* = 20; M4 Δ*hylA* pDCerm, *n* = 10), encapsulated M4 GAS (M4 pHasABC, *n* = 20; M4 Δ*hylA* pHasABC, *n* = 10).

The accumulation of plasmin activity on the cell surface is correlated with invasive disease propensity, enabling GAS to degrade host tissue barriers and spread systemically from the site of localized infection (5). M4 GAS is frequently associated with severe invasive human infections (33, 63), so we assessed the capacity of M4 GAS to acquire plasmin activity. M4 WT and Δ*hylA* accumulated significantly less plasmin than WT M1 GAS (Fig. 7*A*), the serotype most often associated with severe invasive GAS infections (4). Capsule expression in WT M4 and M4 Δ*hylA* improved plasmin activity (Fig. 7*A*), and bacterial survival following a 15-min exposure to freshly isolated human neutrophils *ex vivo* (Fig. 7*B*). However, capsule expression did not enhance the virulence of WT M4 or M4 Δ*hylA* in a mouse model of systemic infection (Fig. 7*C*). The HylA-deficient mutant M4 Δ*hylA* did not display a significant reduction in virulence compared with M4 WT (Fig. 7*C*). Together, these data suggest that capsule expression may not provide a survival advantage for M4 GAS.

## DISCUSSION

After more than a century of research, it is generally accepted that the HA capsule is a major virulence factor, endowing GAS with a protective physical barrier, molecular mimicry, resistance to opsonophagocytosis, and the ability to interact with epithelial cells (6, 7). HA capsule is required for colonization of the upper respiratory tract and production of invasive infections in animal models (10–13), and contributes to human pharyngeal and invasive infections (13, 14, 19). In this investigation, we report that nonencapsulated serotype M4 GAS was a frequent etiologic agent of severe invasive diseases in children. Molecular genetic interrogation of a panel of 17 invasive disease isolates identified 3 distinct PFGE patterns and 2 MLSTs. The majority of isolates were SpeB-negative *covRS* mutants, a distinguishing feature of hypervirulent GAS. All M4 isolates lacked the *hasABC* capsule biosynthesis operon and did not produce detectable HA capsule. Induction of capsule expression in M4 GAS abrogated C4BP binding, an important immune evasion mechanism to subvert complement attack (64), and failed to enhance survival in human blood and virulence in a mouse model of systemic infection. These data demonstrate that the HA capsule is not essential for GAS to cause life-threatening invasive infections in humans.

M4 and M22 GAS serotypes secrete active HylA, an enzyme that degrades the HA present in the GAS capsule and mammalian connective tissues. Other serotypes contain a single nucleotide mutation in *hylA* resulting in Asp to Val substitution at amino acid position 199 in the putative substrate-binding site that completely abolishes HylA enzymatic activity (51). In this study, we demonstrate that capsule production is abolished in M1 strain 5448 expressing the *hylA* gene from M4 GAS. Significantly, transformation of M4 Δ*hylA* with a plasmid expressing the *hasABC* operon from M1 GAS induced capsule expression. These findings demonstrate that M4 GAS has the capacity to synthesize capsule, and that its capsule is stable in the absence of a functional HylA enzyme. However, capsule expression in M4 GAS reduced C4BP binding and neither enhanced whole blood survival nor virulence *in vivo*, suggesting that encapsulation may not provide a survival advantage for this serotype. Mouse and other vertebrate models of GAS infection have significant limitations and drawbacks because GAS is a human-adapted pathogen. Therefore, we cannot exclude the possibility that encapsulated M4 GAS would be more virulent in the human host. The absence of capsule in HylA-expressing serotype M4 and M22 GAS strains (33, 51) suggests a competitive co-evolution between HylA and capsule; however, hyaluronidase expression by encapsulated GAS was reported more than 50 years ago (65–67). Furthermore, some strains of the closely related group C *Streptococcus*, a bacterial pathogen capable of causing human disease (although less frequently than GAS), naturally co-express capsule and a functional hyaluronidase (65, 67).

Highly virulent nonencapsulated strains have been reported for several human bacterial pathogens, including *S. agalactiae* (68), *Haemophilus influenzae* (69), and *Neisseria meningitidis* (70). In the majority of GAS serotypes containing intact *covRS* loci, encapsulation provides significant advantages over non-encapsulation such as molecular mimicry, resistance to phagocytosis, and enhanced adherence to host epithelial cells. The reason for the unsuccessful acquisition of *hasABC* or loss thereof remains unclear; however, M4 GAS may possess additional antiphagocytic factors and adhesins to thwart the host immune response and promote the disease process. Understanding the underlying molecular pathogenesis of nonencapsulated GAS invasive disease may augment the development of a new generation therapeutics and provide better health outcomes in the fight against this globally important human pathogen.

## Supplementary Material

Supplemental Data

## Footnotes

3 The abbreviations used are:

GAS: group A *Streptococcus*
C4BP: C4b-binding protein
*covRS*: control of virulence regulatory system
HA: hyaluronic acid
HylA: hyaluronate lyase
MLST: multilocus sequence typing
PFGE: pulsed-field gel electrophoresis
SpeB: streptococcal pyrogenic exotoxin B
THB: Todd-Hewitt broth
Fg: fibrinogen
nt: nucleotide.
