# Supplementary material for: Mutual Exclusivity of Hyaluronan and Hyaluronidase in Invasive Group A Streptococcus
Source: J Biol Chem. 2014 Sep 29;289(46):32303–15. doi: 10.1074/jbc.M114.602847 (PMC4231703; doi:10.1074/jbc.M114.602847)
Supplement: Supplemental Data [file supp_289_46_32303__index.html]

Mutual Exclusivity of Hyaluronan and Hyaluronidase in Invasive Group A Streptococcus — Mutual Exclusivity of Hyaluronan and Hyaluronidase in Invasive Group A Streptococcus — Hyaluronan and Hyaluronidase in Invasive GAS — Supplemental Data 

# Mutual Exclusivity of Hyaluronan and Hyaluronidase in Invasive Group A *Streptococcus*

## Supplemental Data

**Files in this Data Supplement:**

- Supplementary Tables 1-3 (.pdf, 113 KB) - Supplementary Tables 1-3
