# Supplementary material for: Mutual Exclusivity of Hyaluronan and Hyaluronidase in Invasive Group A Streptococcus
Source: J Biol Chem. 2014 Sep 29;289(46):32303–15. doi: 10.1074/jbc.M114.602847 (PMC4231703; doi:10.1074/jbc.M114.602847)
Supplement: Supplemental Data [file supp_M114.602847_jbc.M114.602847-1.pdf]

SUPPLEMENTARY TABLE 1
Clinical data for M4 GAS strains SP435 and SP436 isolated from brothers

	**SP435**	**SP436**
Hospital admission date	17th February 2001	18th February 2001
Gender	Male	Male
Date of birth	7th July 1999	11th February 1997
Clinical notes	Presented with fever 39.8°C, right hip pain, reluctant to weight bear; tissue and pus from right hip joint grew GAS; bone scan consistent with septic arthritis; erythematous rash on face, trunk and limbs	Presented with fever 40.1°C, right knee pain, lethargy, right knee red and swollen
Blood cultures	Positive for GAS	Positive for GAS
WBC	14,100	25,000
ANC	9,300	22,000
CRP	126 mg/l	218 mg/l
Hemoglobin and platelet counts	Normal	Normal
Blood film	Toxic granulation	Toxic granulation
ESR	94 mm/h	41 mm/h
Treatment	Ceftriaxone, changed to penicillin + clindamycin after positive blood culture result	Flucoxacillin + Ceftriaxone, changed to penicillin after positive blood culture result
Hospital discharge	9th March 2001	2nd March 2001

Abbreviations: ANC, absolute neutrophil count; CRP, C-reactive protein; ESR, erythrocyte sedimentation rate; WBC, white blood cell count

SUPPLEMENTARY TABLE 2

MLST allelic profiles of 17 clinical M4 GAS isolates associated with human invasive disease

M4 isolate	*gki*	*gtr*	*murI*	*mutS*	*recP*	*xpt*	*yqiL*	Sequence type
SP435	5	11	8	5	15	2	1	39
SP436	5	11	8	5	15	2	1	39
SP437	5	11	8	5	15	2	1	39
SP438	5	11	8	5	15	2	1	39
SP439	5	11	8	5	15	2	1	39
SP440	5	11	8	5	15	2	1	39
SP441	5	11	8	5	15	2	1	39
SP442	5	11	8	5	15	2	1	39
SP443	5	11	8	5	15	2	1	39
SP444	5	11	8	5	15	2	1	39
SP445	5	11	8	5	15	2	1	39
SP446	5	11	8	5	15	2	1	39
SP447	5	11	8	5	15	2	1	39
SP448	5	11	8	5	15	2	1	39
SP449	5	11	8	new[a]	15	2	1	tba
SP450	5	11	8	5	15	2	1	39
SP451	5	11	8	new[a]	15	2	1	tba

[a]New allele, closest similarity ~99%

Abbreviations: *gki*, glucose kinase; *gtr*, glutamine transporter protein; *murI*, glutamate racemase; *mutS*, DNA mismatch repair protein; *recP*, transketolase; *xpt*, xanthine phosphoribosyl transferase; *yqiL*, acetyl coenzyme A (acetyl-CoA) acetyltransferase; tba, to be assigned by the *S. pyogenes* MLST database curators (http://spyogenes.mlst.net/).

SUPPLEMENTARY TABLE 3
Distribution of the *hylA* gene among streptococci and other species

Species	Presence/absence	NCBI protein ID
S. pyogenes	+	AAZ51375.1
S. pneumoniae	+	AAK74491.1
S. agalactiae	+	AAN00079.1
S. parauberis	+	AEF25615.1
S. dysgalactiae subsp. *equisimilis*	+	ADX24222.1
S. equi, supsp. *equi*	2 ORFs (truncation)	N/A (pseudogene)
S. equi, subsp. *zooepidemicus*	+	CAW98747.1
S. ictaluri	+	WP_008087497.1
S. suis	+	AEB81651.1
Staphylococcus aureus	+	BAB43294.1
S. thermophilus	–	–
S. uberis	–	–
S. mutans	–	–
Bacillus cereus plasmid	+	AAY60495
Paenibacillus mucilaginosus	+	AFH63711.2

Abbreviations: N/A, not applicable; ORFs, open reading frames; *S.*, *Streptococcus*; +, present; –, absent.
